# Association of Esophageal Cancer Mortality with Municipal Socioeconomic Deprivation Level in Japan, 2013–2017: An Ecological Study Using Nationwide Data

**DOI:** 10.3390/ijerph19095483

**Published:** 2022-04-30

**Authors:** Tasuku Okui, Akie Hirata, Naoki Nakashima

**Affiliations:** Medical Information Center, Kyushu University Hospital, Fukuoka 812-8582, Japan; hirata.akie.006@m.kyushu-u.ac.jp (A.H.); nakashima.naoki.351@m.kyushu-u.ac.jp (N.N.)

**Keywords:** Japan, esophageal cancer, mortality, municipalities

## Abstract

This study aimed to show geographical differences in esophageal cancer mortality in Japan and reveal an association of esophageal cancer mortality with municipal socioeconomic deprivation level. Esophageal cancer mortality data in the Vital Statistics from 2013 to 2017 for each municipality and corresponding population data were analyzed. The deprivation level was derived from the municipal socioeconomic variables by principal component analysis. Municipalities were classified into five quintiles based on the deprivation level, and an association between the level and esophageal cancer mortality was evaluated using a Bayesian spatial model. As a result of regression analysis, the relative risk of esophageal cancer mortality tended to become larger as the deprivation level increased irrespective of sex, and the relative risk of esophageal cancer mortality was significantly higher in quintile 5 (most deprived) than quintile 1 (least deprived) among men and women. These results suggest that the deprivation level increases the risk of esophageal cancer mortality in Japan.

## 1. Introduction

Esophageal cancer is the eighth most common cancer type worldwide, and is known to be the sixth leading cause of cancer-related mortality [1,2]. Esophageal cancer has two major histologic types, namely, esophageal adenocarcinoma and esophageal squamous cell carcinoma. Esophageal adenocarcinoma is more common in Western countries; however, squamous cell carcinoma is the predominant histologic type in Japan [3,4].

In Japan, age-standardized mortality rates of esophageal cancer are decreasing over the last few decades in both sexes. In addition, the mortality rate of esophageal cancer is relatively low compared with other major cancer types in Japan. However, prevention is needed because the age-standardized incidence rate is not decreasing [5,6], and the 5- and 10-year survival rates of patients with esophageal cancer are relatively low compared with other cancer types [7].

In other countries, some epidemiological studies have shown that the incidence, mortality, and survival rates of esophageal cancer vary depending on socioeconomic status [8,9,10,11]. Particularly, some previous studies have focused on the differences in esophageal cancer mortality depending on the areal socioeconomic deprivation level, and disparities in esophageal cancer mortality depending on socioeconomic deprivation level have been shown [8,10].

In Japan, a study using the nationwide data reported an association of socioeconomic deprivation level with the mortality rate for some cancer types [12], and the cancer mortality risk tended to become higher as the deprivation elevated. Additionally, previous studies have revealed a disparity in the survival rates of major cancer types depending on socioeconomic status, such as income or occupation [13,14,15], and the lower occupational class was associated with poor prognosis. Contrastingly, the standardized mortality ratio (SMR) of major types of cancer tended to be low in areas where the socioeconomic position was low among women [16]. Therefore, the relationship between socioeconomic status and cancer mortality varies depending on socioeconomic status indicators in Japan. In contrast, few previous studies investigated an association of mortality with socioeconomic status or socioeconomic deprivation level for esophageal cancer in Japan. Only one case–control study investigated the association [17], and to our knowledge, no study has investigated the association of municipal deprivation socioeconomic level with mortality using the nationwide statistical data. Moreover, the difference in the SMR for esophageal cancer has not been revealed using municipal-level data in Japan, and areas with high SMR are unknown.

This study aimed to reveal the association of esophageal cancer mortality with a municipal socioeconomic deprivation level in Japan using recent Vital Statistics. Specifically, the municipal socioeconomic deprivation level from municipal socioeconomic characteristics was derived by principal component analysis, and an association of esophageal cancer mortality with the deprivation level was shown by a Bayesian spatial model.

Data used in this study were shown in the Materials and Methods section. Additionally, the process of deriving the municipal socioeconomic deprivation level and statistical analysis methods used for investigating the association were explained. The Results section showed geographical differences in SMR for esophageal mortality in Japan and the results of the Bayesian spatial model. The Discussion presents possible reasons for the association of esophageal cancer mortality with the deprivation level and study implications and limitations.

## 2. Materials and Methods

Mortality data of esophageal cancer obtained from the Vital Statistics in Japan from 2013 to 2017 for each municipality were analyzed [18]. Individuals diagnosed with C15 (International Classification of Diseases, 10^th^ Revision, codes) corresponding to esophageal cancer were enrolled. Population data of each municipality for the corresponding years were obtained from the survey of the population, demographics, and household number based on the basic resident register [19].

To determine the socioeconomic deprivation level, some characteristics of municipalities in 2015 were used. This study used the following data: proportion of fatherless households (Number of fatherless households per number of total households), proportion of low educational level (Number of elementary school graduates and junior high school graduates per number of persons aged 15 years or more), proportion of unemployed persons (Number of unemployed persons per labor force population), taxable income per capita, proportion of divorced persons (Number of divorced persons per total population over 15 years old), proportion of laborers (Number of laborers per labor force population), and proportion of households living in owner-occupied housing (Number of households living in owner-occupied housing per number of total private households). Data except for taxable income were obtained from the Census [20,21], and data on taxable income were obtained from the survey on conditions of municipal taxation by the Ministry of Internal Affairs and Communications [20]. Data on educational level cannot be obtained in the Census in 2015, and we used the data in 2010 for the proportion of low educational level. Map data of Japan were obtained from the digital national land information (administrative area data) published by government and processed by the authors [22].

In the statistical analysis, the esophageal mortality rate of all of Japan for each age group were calculated. Using the overall esophageal mortality rates in Japan and age group-specific population for each municipality, the expected esophageal mortality for each municipality was calculated. Then, the SMR of esophageal mortality was calculated. SMR was calculated by empirical Bayes method using DCluster (https://cran.r-project.org/web/packages/DCluster/DCluster.pdf, accessed on 29 April 2022) [23]. The SMR of each municipality was mapped in the map of Japan. In addition, we identified municipalities whose SMRs were particularly high. Moreover, we tested spatial autocorrelation of SMR by Moran’s I test using spdep (https://cran.r-project.org/web/packages/spdep/spdep.pdf, accessed on 29 April 2022).

Areal socioeconomic deprivation level is often derived by summarizing multiple socioeconomic variables [24,25,26,27,28], and principal component (PC) analysis is frequently used for the derivation [24,25]. In a method using PC analysis, socioeconomic variables are summarized into few PCs, and the PC score is used as a socioeconomic deprivation level [25]. When calculating municipal socioeconomic deprivation level, all the variables were standardized.

Based on municipal socioeconomic deprivation level, we classified municipalities into five quintiles. Then, an ecological study was conducted using each municipality as one observation. Specifically, we used a Bayesian spatial model for investigating an association between municipal deprivation socioeconomic level and esophageal cancer mortality [12,29]. A Bayesian spatial model with spatial random effects following conditional autoregressive distribution was used in the analysis to consider spatial correlation among municipalities [29]. The model is called Besag-York-Mollié model [30]. The observed number of esophageal cancer mortality was supposed to follow the Poisson distribution in the model. Therefore, municipalities who are not adjoining any other municipalities were excluded in the analysis. The observed and expected numbers of esophageal cancer deaths were used as the outcome and offset, respectively, in the Bayesian spatial model. All statistical analyses were conducted using R4.1.3 (https://www.R-project.org/, accessed on 29 April 2022).

## 3. Results

**Figure 1:**
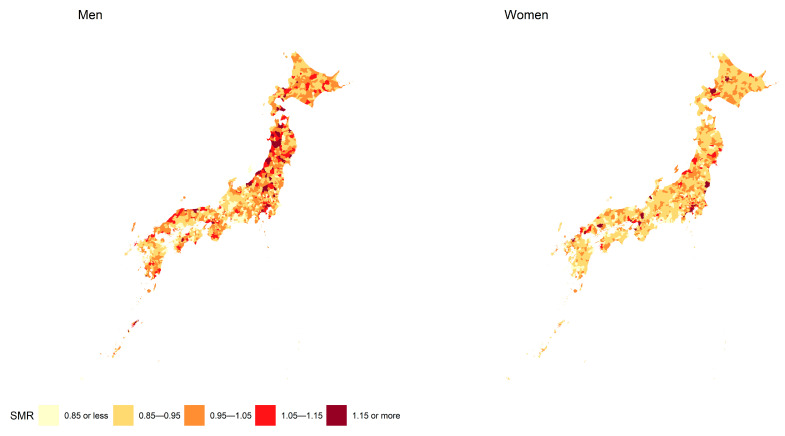
Geographic distribution of SMR of esophageal cancer for men and women. The left map indicates SMRs of men, and the right one indicates those of women.

Figure 1 shows the geographic distribution of the SMR of esophageal cancer for men and women. Municipalities whose SMRs were in 1.05–1.15 or above 1.15 were observed more often in men. Moran’s I statistics for men was 0.124 (p value < 0.001) and that for women was 0.097 (p value < 0.001). Therefore, a spatial autocorrelation existed among the SMRs, and a spatial model was suggested to be useful for the association analysis.

Table 1 shows the top 10 municipalities with high SMR of esophageal cancer. Wards in Tokyo, Osaka city, and Fukuoka city, which are among the most urbanized areas in Japan, appeared in the top 10 municipalities, particularly in women.

Table 2 shows basic characteristics of municipalities used in the analysis. Overall, 1687 municipalities, which are adjacent to other municipalities, were used in the analysis.

Table 3 shows the result of the PC analysis. The variable loading indicates a correlation coefficient between each variable and a score of the PC. The proportions of variance explained for the first PC and second PC were particularly large. The first PC can be interpreted the socioeconomic status among individuals because taxable income per capita and the proportion of households living in owner-occupied housing were positively correlated with the score and those of other variables were negatively correlated with the score. The higher the score of the first PC, the higher the level of socioeconomic status among individuals. Therefore, the score of the first PC was used as municipal socioeconomic deprivation level in the following analysis. The score of the first PC is calculated by the following equation, and standardized values are used for all the variables.

Score of the first PC = −0.517 × proportion of fatherless households −0.110 × proportion of low educational level −0.470 × proportion of unemployed persons +0.293 × taxable income per capita −0.591 × proportion of divorced persons −0.201 × proportion of laborers +0.152 × proportion of households living in owner-occupied housing.

We classified municipalities into five quintiles based on the score of the first PC, and used the quintiles as an explanatory variable in the Bayesian spatial model.

Table 4 shows the relative risk and 95% credible interval (CI) of esophageal cancer mortality obtained using the Bayesian spatial model. The analysis was conducted by sex. Quintile 1 is the least deprived municipalities, and their first PC scores were the highest among municipalities. Specifically, the five quintiles were used as a factor variable, and quintile 1 (least deprived) was reference. Therefore, relative risk of each quintile to quintile 1 was calculated. Relative risk that is higher than 1 for certain quintile indicates a higher risk of esophageal cancer mortality of the quintile than quintile 1. The relative risk tended to become larger as the deprivation level increased irrespective of sex, and the relative risk of esophageal cancer mortality was significantly higher in quintile 5 (most deprived) compared with quintile 1 in males and females.

## 4. Discussion

To the best of our knowledge, this is the first study that revealed the geographic distribution of SMR of esophageal cancer in Japan using municipal-level data. Figure 1 suggested the presence of clusters of municipalities with a high relative risk of mortality, particularly among men, which led us to consider a regression model taking into account the spatial correlation between municipalities. Municipalities whose SMR were high were observed more often in men compared with women. Many municipalities had relatively low mortality of women with esophageal cancer, and we found that differences among municipalities were barely observed in women compared with men. In addition, municipalities with the highest score of PC indicating socioeconomic status among individuals had the lowest risk of esophageal cancer mortality in men and women. Similarly, the SMRs of some wards in Tokyo were particularly high in women. We discussed possible reasons for the association.

The incidence rate of esophageal cancer was possibly different depending on the socioeconomic status of individuals. Major lifestyle-related risk factors for esophageal cancer, particularly for esophageal squamous cell carcinoma, are smoking and alcohol drinking [4,31,32]. In Japan, the smoking prevalence is higher in persons with low socioeconomic status [33]. In addition, persons with low educational attainment tended to become problematic heavy alcohol drinkers [34]. Poor dietary habits are other risk factors, and an epidemiological study in Japan showed that fruit and vegetable consumption reduces the incidence of esophageal squamous cell carcinoma [35]. In Japan, higher household income and education levels were significantly associated with higher rates of eating vegetables [36], and socioeconomic status was shown to be associated with habits of eating vegetables [37]. Differences in these health behaviors among regions may lead to the variance in the incidence of esophageal cancer.

Moreover, there might be differences in the survival rate of patients with esophageal cancer depending on socioeconomic status of individuals. In other countries, the survival rate of esophageal cancer differed depending on the socioeconomic level [9,10]. According to a population-based study in France, the reason for the phenomenon is uncertain, but the disparities cannot be explained by factors such as differences in access to care and cancer spread [10]. A study in China reported that socioeconomic status was associated with health care delay, tumor stage, and treatment modalities [38]. In Japan, patients with higher income were more often diagnosed with early-stage disease [39], and stages at the time of diagnosis for esophageal cancer may differ depending on the socioeconomic deprivation level. In Japan, the participation rate in cancer screening is largely different depending on the socioeconomic status [40,41], and higher income is related to a higher rate of health service utilization [42,43].

The SMRs of urbanized areas were particularly high in women. The relationship between esophageal cancer and urbanization varies depending on the country. A higher incidence and mortality of esophageal cancer in rural areas than urban areas have been shown in China [8,44], and regional differences in factors (e.g., educational level, smoking and drinking habits, and water pollution) are possible reasons. In Japan, smoking prevalence in older birth cohorts is higher in urban areas than nonurban areas for women [45], and it may be related to an association between urbanization and esophageal cancer mortality. Additionally, according to a study conducted in the 1990s against young mothers in Japan, the proportion of current alcohol drinkers was greater in a more-urbanized area than that in a less-urbanized area [46], and exposure to alcohol drinking may differ depending on regions among middle and old aged women at the present.

The results of this study suggested that the areal socioeconomic level or neighborhood environment is associated with the mortality rate of esophageal cancer in Japan. Furthermore, socioeconomic status may affect health behaviors and social and medical resources of areas. Therefore, not only the decrease in disparity in social and medical resources among regions, but also a health promotion movement against municipality with “socioeconomically deprived areas” is needed to ease disparity. Thus, future studies need to verify whether municipal socioeconomic deprivation level affects both the incidence and survival rates of esophageal cancer. A further epidemiological study is needed to scrutinize reasons for the association.

This study has some limitations. First, this is an ecological study, and an epidemiological study using individual data is warranted to verify the results. Secondly, we could not obtain data on the mortality rate of esophageal cancer by histologic types, while esophageal squamous cell carcinoma is the predominant type in Japan [3]. Thirdly, there are several methods for deriving municipal socioeconomic level, and it will be meaningful to use other methods in the future. By contrast, the strength of this study was related to the use of nationwide vital statistics data, so the result of this study represents the trend of all of Japan.

## 5. Conclusions

We revealed the association of esophageal cancer mortality with municipal socioeconomic deprivation level in Japan using vital statistics in recent years. As a result of spatial regression analysis, the relative risk of esophageal cancer mortality tended to become larger as the deprivation level increased irrespective of sex. These results suggest that municipal socioeconomic deprivation level increases the risk of esophageal cancer mortality in Japan. Preventive measures are particularly needed in those areas, and a further epidemiological study investigating differences in health status or incidence and survival rates of esophageal cancer depending on areas is needed to scrutinize reasons for the association.

## Figures and Tables

**Table 1:** Top 10 municipalities with high SMR of esophageal cancer.

	Men		Women	
Rank	Municipality Name (Prefecture Name)	SMR	Municipality Name (Prefecture Name)	SMR
1	Otaru city (Hokkaido)	1.437	Arakawa ward (Tokyo)	1.560
2	Taito ward (Tokyo)	1.437	Osaka city (Osaka)	1.448
3	Kita ward (Tokyo)	1.403	Shinagawa ward (Tokyo)	1.404
4	Daisen city (Akita)	1.381	Odawara city (Kanagawa)	1.379
5	Akita city (Akita)	1.373	Katsushika ward (Tokyo)	1.375
6	Nakano ward (Tokyo)	1.333	Minato ward (Tokyo)	1.365
7	Yugawara town (Kanagawa)	1.321	Nakano ward (Tokyo)	1.351
8	Kesenuma city (Miyagi)	1.319	Suginami ward (Tokyo)	1.344
9	Noshiro city (Akita)	1.310	Fukuoka city (Fukuoka)	1.338
10	Joetsu city (Niigata)	1.307	Nishitokyo city (Tokyo)	1.326

SMR, standardized mortality ratio.

**Table 2:** Basic characteristics of municipalities used in the analysis.

Municipal Characteristics	Median (Interquartile Range)
	(*N* = 1687)
Proportion of fatherless households	1.4 (1.1–1.7)
Proportion of divorced persons	5.0 (4.3–5.8)
Proportion of persons with low educational level	22.7 (16.3–30.4)
Proportion of laborers	7.3 (6.5–8.4)
Proportion of unemployed persons	3.9 (3.3–4.7)
Taxable income per capita (1000 yen)	1103.3 (931.3–1293.0)
Proportion of households living in owner–occupied housing	74.7 (65.8–83.1)
Male esophageal cancer mortality rate *	15.8 (11.9–20.7)
Female esophageal cancer mortality rate *	2.5 (0.0–4.1)

* Number of deaths per 100,000 person-years.

**Table 3:** Result of the principal component analysis. (Up to the PC 4).

Variables and variance explained	Variable Loadings			
	PC 1	PC 2	PC 3	PC 4
Proportion of fatherless households	−0.787	0.375	−0.110	0.199
Proportion of divorced persons	−0.899	0.088	0.119	0.194
Proportion of persons with low educational level	−0.167	−0.854	0.195	0.224
Proportion of laborers	−0.306	−0.578	0.558	−0.450
Proportion of unemployed persons	−0.715	0.177	−0.368	−0.489
Taxable income per capita	0.446	0.764	0.223	−0.205
Proportion of households living in owner–occupied housing	0.231	−0.690	−0.600	−0.120
Square root of eigenvalue	1.521	1.518	0.960	0.791
Proportion of variance explained	0.330	0.329	0.132	0.089
Cumulative proportion of variance explained	0.330	0.660	0.791	0.881

PC, Principal component.

**Table 4:** The relative risk (and 95% CI) of esophageal cancer mortality obtained by the Bayesian spatial model.

	Men	Women
Quintiles based on the deprivation level	Relative risk (95% CI)	Relative risk (95% CI)
Quintile 1 (Least derived)	Reference	Reference
Quintile 2	1.032 (0.989, 1.080)	0.979 (0.895, 1.074)
Quintile 3	1.069 (1.023, 1.119)	1.062 (0.970, 1.167)
Quintile 4	1.081 (1.030, 1.140)	1.101 (0.997, 1.217)
Quintile 5 (Most derived)	1.157 (1.095, 1.228)	1.269 (1.129, 1.423)

CI, credible interval; PC, principal component.

## Data Availability

All the data used in this study are from government statistics, and are publicly available. The data sources are written in the References.
